# HPV self-sampling among long-term non-attenders to cervical cancer screening in Norway: a pragmatic randomised controlled trial

**DOI:** 10.1038/s41416-022-01954-9

**Published:** 2022-08-23

**Authors:** Gunvor Aasbø, Ameli Tropè, Mari Nygård, Irene Kraus Christiansen, Ingrid Baasland, Grete Alrek Iversen, Ane Cecilie Munk, Marit Halonen Christiansen, Gro Kummeneje Presthus, Karina Undem, Tone Bjørge, Philip E. Castle, Bo T. Hansen

**Affiliations:** 1 Department of Research, Cancer Registry of Norway, Oslo, Norway; 2 Department of Interdisciplinary Health Sciences, Institute of Health and Society, University of Oslo, Oslo, Norway; 3 Section for Cervical Cancer Screening, Cancer Registry of Norway, Oslo, Norway; 4 National HPV Reference Laboratory, Department of Microbiology and Infection Control, Akershus University Hospital, Lørenskog, Norway; 5 Department of Public Health and Nursing, Norwegian University of Science and Technology, Trondheim, Norway; 6 Department of Obstetrics and Gynaecology, Haukeland University Hospital, Bergen, Norway; 7 Department of Obstetrics and Gynaecology, Sørlandet Hospital Kristiansand, Kristiansand, Norway; 8 Department of Obstetrics and Gynaecology, Stavanger University Hospital, Stavanger, Norway; 9 National Institute of Occupational Health, Oslo, Norway; 10 Department of Global Public Health and Primary Care, University of Bergen, Bergen, Norway; 11 Division of Cancer Prevention, National Cancer Institute, National Institutes of Health, Rockville, MD USA; 12 Divisions of Cancer Epidemiology and Genetics, National Cancer Institute, National Institutes of Health, Rockville, MD USA; 13 Department of Infection Control and Vaccine, Norwegian Institute of Public Health, Oslo, Norway

**Keywords:** Preventive medicine, Population screening, Epidemiology

## Abstract

**Background:**

Cervical cancer screening participation is suboptimal in most settings. We assessed whether human papillomavirus (HPV) self-sampling may increase screening participation among long-term non-attenders in Norway.

**Methods:**

A pragmatic randomised controlled trial with participation as the primary outcome was initiated in the national cervical screening programme in March 2019. A random sample of 6000 women aged 35–69 years who had not attended screening for at least 10 years were randomised 1:1:1 to receive either (i) a reminder to attend regular screening (control), (ii) an offer to order a self-sampling kit (opt-in) for HPV testing or (iii) a self-sampling kit unsolicited (send-to-all) for HPV testing.

**Results:**

Total participation was 4.8%, 17.0% and 27.7% among control, opt-in and send-to-all (*P* < 0.0001; participation difference (%) send-to-all vs. control: 22.9 (95%CI: 20.7, 25.2); opt-in vs. control: 12.3 (95%CI: 10.3, 14.2); send-to-all vs. opt-in: 10.7 (95% CI: 8.0, 13.3)). High-risk HPV was detected in 11.5% of self-samples and 9.2% of clinician-collected samples (*P* = 0.40). Most women (92.5%) who returned a positive self-sample attended the clinic for triage testing. Of the 933 women screened, 33 (3.5%) had CIN2 + (1.1%, 3.7%, 3.8% among control, opt-in, and send-to-all, respectively), and 11 (1.2%) had cervical cancer (0%, 1.2%, 1.3% among control, opt-in, send-to-all, respectively).

**Conclusion:**

Opt-in and send-to-all self-sampling increased screening participation among long-term, higher-risk non-attenders.

**Clinical trial registration:**

ClinicalTrials.gov NCT03873376.

## Background

Screening has reduced cervical cancer incidence [1] and mortality [2] substantially in Norway. However, coverage of the national cervical cancer screening programme has stagnated at a suboptimal 70% [3], and cervical cancer incidence has increased by 14% from the period 2009–2013 to 2014–2018 [3]. Under- or unscreened women have an increased risk of cervical cancer [4] and being diagnosed at an advanced stage [5]. Thus, interventions that improve screening participation would benefit women’s health.

Approximately 17% of women aged 35–69 years in Norway have not been screened for at least 10 years [3]. In Sweden, the detection of high-grade cervical abnormalities among women who had not been screened during the last 10 years was considerably higher than in the general screening population [6], which highlights the need for improved interventions among long-term non-attenders. Important reasons for non-attendance include procrastination, embarrassment, fear of pain and previous negative experiences with the gynaecological exam or a history of sexual abuse [6, 7], as well as practical barriers experienced in everyday life [8]. Non-attendance among Norwegian women is also associated with having a male/foreign/young general practitioner (GP) [9], lacking awareness of the recommended screening interval [10], and lower socioeconomic or migrant background [11]. Self-sampling for human papillomavirus (HPV) testing, which the women can perform themselves at home, may reinforce the importance of screening, empower women, and mitigate some of the barriers associated with attending a gynaecological screening exam.

HPV infection is a necessary cause of cervical cancer [12]. Persistent infection with high-risk HPV (hrHPV) may, through intermediate precancerous stages, lead to cancer [13]. Moreover, when cervical cancer is detected at a late stage, effective treatment is limited and prognosis poor [14]. When used with HPV assays based on polymerase chain reaction (PCR), self-sampling and HPV testing identifies women with cervical precancer and cancer, cervical intraepithelial neoplasia grade 2 or more severe diagnoses (CIN2+), with similar accuracy as clinician-collected samples [15, 16]. Studies also show that self-sampling has a high acceptability among women and is preferred over clinician sampling [17]. Send-to-all strategies, where women receive a self-sampling kit unsolicited, proved to increase screening participation of under-screened women in a relatively recent meta-analysis [15], and in Norway [18]. Opt-in strategies, where women must request a self-sampling kit, have generally not been found to be more effective than invitation letters for increasing screening participation. The effect of self-sampling on participation varies between studies, especially for opt-in strategies, and warrants more studies [15].

Studies on the effect of HPV self-sampling on participation among under-screened women often include women only slightly overdue for screening, who may be easier to engage than long-term non-attenders, while few randomised controlled trials have by design specifically targeted long-term non-attenders [6, 19–22]. It is important to gain more knowledge on how secondary cancer prevention among long-term non-attenders may be improved because they may be at a relatively high risk for CIN2+.

### Aim

The primary aim of this study was to compare cervical cancer screening participation among women in Norway who have not attended screening for at least 10 years, who received either a standard reminder letter to attend screening in a clinic (control arm), an offer to order an HPV self-sampling kit (opt-in arm), or were sent an unsolicited HPV self-sampling kit (send-to-all arm). As secondary endpoints, we estimated corresponding hrHPV positivity rates and occurrence of CIN2+ among the long-term non-attenders who participated in screening, and attendance to follow-up.

## Materials and methods

### Cervical screening in Norway

The Cancer Registry of Norway (CRN) is responsible for the national cervical cancer screening programme (CervicalScreen Norway), and invites women aged 25–69 years to attend screening. The programme is currently transitioning from cytology every 3 years to HPV primary screening every 5 years for women aged 34–69 years. CervicalScreen Norway uses a centralised invitation procedure and issues standard open reminder letters to women who have not been registered with a screening test during the recommended interval. A second reminder is issued after one year if a woman is still not registered with a test result. These reminders encourage women to schedule an appointment with their GP or a gynaecologist for a screening test. All cytology and HPV screening results, as well as any associated histology results, are registered in the CervicalScreen Norway database. All records are associated with the personal identity number unique to each Norwegian resident.

### Study population and design

The target population was women residing in the counties of Hordaland, Rogaland, Sør-Trøndelag and Vest-Agder who had not participated in the screening programme for the last 10 years. These regions consist of mixed rural and urban areas and cover about one-third of the Norwegian population. During the study, primary screening was offered by HPV test in Hordaland, Rogaland and Sør-Trøndelag, and by cytology in Vest-Agder. A priori power calculations showed that we needed at least 1417 women in each intervention arm to detect a difference of 5 percentage points between the control arm and the intervention arms with 90% power. Using the CervicalScreen Norway database, we identified 28,125 women who were eligible for the study, of which 6000 (21.3%) were randomly selected for invitation to the study. The randomly selected women were individually randomised 1:1:1 into the control, opt-in or send-to-all intervention arm without further restrictions. The eligible women were unaware of the randomisation. Blinding was not possible due to the nature of the interventions. Randomisation procedures were performed using the sample function in Stata by a programmer who was not involved in the conduct of the trial. Invitations were sent during March–August 2019.

**Fig. 1:**
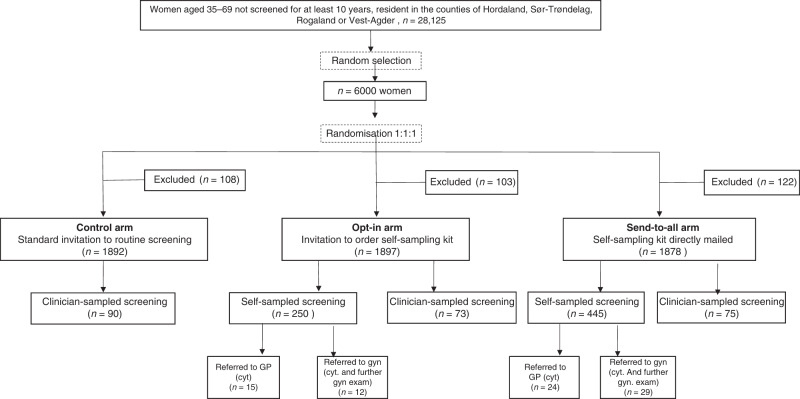
Study flowchart. Excluded women were either not reached, not eligible or declined to participate in the study.

A total of 333 (5.6%) invited women, similarly distributed by intervention arm; 108 (5.4%) in the control arm, 103 (5.1%) in opt-in arm, and 122 (6.1%) in the send-to-all arm; *P* = 0.4), were excluded from the study due to: (i) incorrect address, (ii) active refusal to participate in the study, or (iii) ineligibility for the study (living abroad/had prior hysterectomy/had a screening test between study sampling and invitation) (see Fig. 1). The trial was registered at ClinicalTrials.gov on March 8, 2019 (NCT03873376).

### Invitation letters and information

Women in the control arm received a CervicalScreen Norway reminder letter to attend the regular screening (i.e., encouraging the women to schedule an appointment with their GP). The letter also stated that they were randomly selected to participate in a study aiming to improve cervical cancer prevention. The letter included no information about self-sampling.

Women in the opt-in arm received an invitation letter to order a self-sampling kit. The women could order by mail, e-mail, or through a webpage. The invitation included a unique order code, information about the study, a reply slip, and a prepaid envelope to return the order. A dedicated webpage for ordering a self-sampling kit was available in Norwegian, English, French, Spanish, Polish, Turkish and Arabic. Each order code could only generate one web order.

A self-sampling kit was mailed unsolicited to women in the send-to-all arm, together with the study invitation letter. The content in the opt-in and send-to-all invitation letters was the same (apart from the description of the ordering procedure in the opt-in letter) and included information that the women alternatively could schedule a regular screening appointment with their GP if they did not want to use the self-sample.

Similar to regular CervicalScreen Norway reminders, all invitation letters contained general screening information. The letters were on CervicalScreen Norway stationary and were signed by the CervicalScreen Norway head. In addition, letters contained study-specific information, a link to the study webpage, and information that they could refrain from participating in the study. All invitation letters were in Norwegian, except for an English reference to a webpage. The study webpage was available in Norwegian and English and included a link to an instructional video on how to collect a self-sample with options for Norwegian or English audio, and English, French, Spanish, Polish, Turkish or Arabic subtitles.

### The HPV self-sampling kit

The kit included the self-sampling device with user instructions, a sealable plastic bag, a prepaid return envelope addressed to the laboratory, and an information leaflet. The Evalyn® Brush (dry brush) (Rovers Medical Devices B.V, Oss, Netherlands) was used for self-sampling in this study. The self-sampling device had a hidden radio frequency identification chip containing a pseudonymized number unique to each invited woman and had no visible personal information. Women returned the self-sample in the plastic bag and mailed it in the return envelope. Women who had not ordered/returned a self-sample within 3 weeks received one reminder letter to order/return the self-sample. The self-sampling kit was free of charge for the women.

### Processing of the screening tests

All self-samples were analysed at the Norwegian HPV reference laboratory at Akershus University Hospital. Soon after receipt at the laboratory, the brush tip was removed from the self-sampling device, transferred to a vial with 4.5 mL ThinPrep PreservCyt and stored at room temperature for at least 24 h. During storage, tubes with the brush tip were vortexed three times for 15 s to ensure proper dissolution of the sample material. The samples were tested for hrHPV using the Cobas® 4800 HPV test (Roche Molecular Systems, Inc, Branchburg, NJ), which individually reports HPV16 and HPV18 results, in addition to a pooled result of the 12 hrHPV types 31, 33, 35, 39, 45, 51, 52, 56, 58, 59, 66 and 68. Beta-globin is included as internal quality control in a fourth channel. Samples with an invalid test result at the first run were retested, which gave a valid test result in every case. Analysis was completed within 21 days after receipt for all samples.

Clinician-taken screening samples were processed according to CervicalScreen Norway guidelines [23]. They were collected by gynaecological examination at a GP or a gynaecologist, transported to the lab in ThinPrep PreservCyt and subjected to primary screening by Cobas® 4800 HPV testing (*N* = 218, 91.6%) or cytology (*N* = 20, 8.4%).

### Clinical management

All women who returned a self-sample were informed about their test result per ordinary mail within 6 weeks after receipt of the self-sample in the lab. Women with an HPV-negative result were encouraged to continue attending the regular cervical cancer screening programme at the recommended interval. Women with a hrHPV-positive self-sample received the result together with a pre-booked triage appointment with a physician. To address the feasibility and performance of alternative triage strategies that could be implemented in a screening programme, women were sequentially allocated to triage at their GP or a gynaecologist practising in the largest city in their county. The notification letter to women in each group was identical. Physicians were informed that the patient had tested positive for HPV after participating in a study offering HPV self-sampling to long-term non-attenders. Women referred to GP triage underwent cytology, while women referred to gynaecologist triage underwent further gynaecological examination, which always included cytology and colposcopy, and biopsy if deemed necessary. To simulate a routine healthcare setting, the women paid the deductible for the GP or the gynaecologist appointment themselves (about 30€ and 60€, respectively), as they would in the CervicalScreen Norway. Management after the scheduled triage visit of women who had a positive hrHPV self-sample was not specific for the study and thus follow national guidelines [23]. Similarly, women who had a clinician-collected screening sample (irrespective of treatment arm), or who had a triage cytology with another physician than she was allocated to in the study were managed outside the study according to the CervicalScreen Norway screening algorithm (Supplementary Fig 1). Reflex triage on the screening sample is standard procedure for both primary screening settings (HPV and cytology), thus HPV/cytology triage does not require a separate visit to a clinic. Women who are HPV16/18-positive are referred to colposcopy with biopsy if the cytology is low- or high grade or to a new HPV test in 12 months if the cytology is normal. Women who are positive for other hrHPV types are referred to colposcopy with biopsy if cytology high grade, to a new HPV test in 12 months if cytology low-grade, or to a new HPV test in 24 months if cytology is normal. Cytology is reported according to the Bethesda system.

### Registry data collection

Data regarding clinician-collected screening tests (including triage tests taken outside the study) was taken from the CervicalScreen Norway database. Data on histological outcomes from colposcopy referrals were taken from the CervicalScreen Norway and the CRN databases. Dates were delivered by month and year. If several histological diagnoses were available for the same women, we only considered the most severe diagnosis. The last registry linkage was performed in December 2020, which allowed at least 16 months of registry follow-up after the study invitation. Histology diagnoses were interpreted according to WHO guidelines [24]. Income data were collected by linkage to Statistics Norway.

### Statistical analysis

Screening participation was defined as returning a valid self-sample or having a clinician-collected screening test within 6 months after receipt of the invitation letter. Attendance to triage was assessed among women with a hrHPV-positive self-sample and was defined as attending at the allocated physician (i.e., GP or study gynaecologist) or an unallocated physician within 6 months of notification of a positive self-sample. Women who returned a hrHPV-positive self-sample and who were not registered with a cytology test by 3 months following the scheduled appointment received a reminder from the CervicalScreen Norway encouraging them to order an appointment at their GP. Screening participation among women in the control arm was only possible by attendance to a clinician. For each self-sampling arm, we present data separately and in total for women who self-sampled and women who had a clinician-collected screening test. The main analyses of participation, which is the trial´s primary outcome measure, are intention-to-treat (i.e., by the total estimates). We also provide per protocol analyses for the participation differences as a supplement. We present numbers and proportions of screening participation by intervention arm (control, opt-in, send-to-all) overall, by age groups (36–45, 46–55, 56–65, 66–69) and by screening history (time since last screening test 10–15 years, 16–28 years, never). Differences in screening participation among intervention arms are presented as absolute participation differences (APD, percentage points) and as relative participation differences (RPD, relative risks) with associated 95% confidence intervals (95% CI). Further, we present numbers and proportions of hrHPV-positive screening tests by intervention arm. We present attendance to triage by self-sampling arm and by mode of triage (i.e., at a gynaecologist or GP) for women with a hrHPV-positive self-sample. Since positive clinician-collected screening tests do not require a separate triage visit, we present attendance to the CervicalScreen Norway recommended follow-up after a positive reflex triage test for women in the control arm and women in the self-sampling arms who screened clinically. Numbers and proportions of histologically verified high-grade lesions are presented by intervention arm. To investigate the diagnostic yield by mode of triage, we also present the histologically verified high-grade lesions separately for women triaged by study gynaecologists, women triaged by study GPs, and women who had a clinician-collected screening test or attended triage outside the study (i.e., women not managed by a study gynaecologist/GP). For comparisons of counts and proportions, *P* values refer to chi-squared tests, or the Fisher´s exact test for observed counts <5. For comparisons of central tendency, *P* values refer to the Mann–Whitney *U* test. Homogeneity of variance was assessed by Levene’s test. All tests were two-sided and *P* values < 0.05 were considered statistically significant. Analyses were performed using Stata version 17MP or R version 3.5.3.

## Results

All baseline characteristics addressed in the study cohort were similarly distributed by the study arm (Table 1). The mean age was 54.5, 54.4 and 54.1 years among women in the control, opt-in and send-to-all arm. Similar frequency distributions in each study arm were observed for categories of age (*P* = 0.94), time since last screen (*P* = 0.99), county of residence (*P* = 0.96) and income (*P* = 0.28) (Table 1).

**Table 1:** Baseline characteristics of the women in the study population.

Characteristic	Total, all arms, *n* (%)	Control group, *n* (%)	Opt-in, *n* (%)	Send-to-all, *n* (%)
Total invited	5667 (100)	1892 (100)	1897 (100)	1878 (100)
Age (years)				
36–45	1318 (23.3)	435 (23.0)	433 (22.8)	450 (24.0)
46–55	1520 (26.8)	497 (26.3)	520 (27.4)	504 (26.8)
56–65	1961 (34.6)	663 (35.0)	653 (34.4)	646 (34.4)
66–69	868 (15.3)	298 (15.7)	292 (15.4)	278 (14.8)
Mean age (standard deviation)	54.3 (9.9)	54.5 (10.0)	54.4 (9.8)	54.1 (9.9)
Time since last screening test				
10–15 years	1786 (31.5)	599 (31.7)	590 (31.1)	599 (31.9)
Over 15 years	1950 (34.4)	650 (34.3)	658 (34.7)	642 (34.2)
Never screened	1931 (34.1)	644 (34.0)	650 (34.2)	637 (33.9)
County				
Hordaland	2106 (37.1)	707 (37.3)	710 (37.4)	689 (36.7)
Rogaland	1705 (30.1)	568 (30.0)	563 (29.7)	574 (30.6)
Trøndelag	1217 (21.5)	415 (21.9)	405 (21.3)	399 (21.2)
Vest-Agder	639 (11.3)	203 (10.7)	220 (11.6)	216 (11.5)
Total income (tertiles)^a^				
Low	1877 (33.3)	622 (33.0)	632 (33.5)	623 (33.4)
Medium	1878 (33.3)	610 (32.4)	659 (34.9)	609 (32.7)
High	1877 (33.3)	650 (34.5)	595 (31.5)	632 (33.9)

^a^Total annual income (wage, welfare payment and capital income) from the most recent Statistics Norway registration during 2014–2018, by tertiles of the total study population. Data missing for 37 women.

**Fig. 2:**
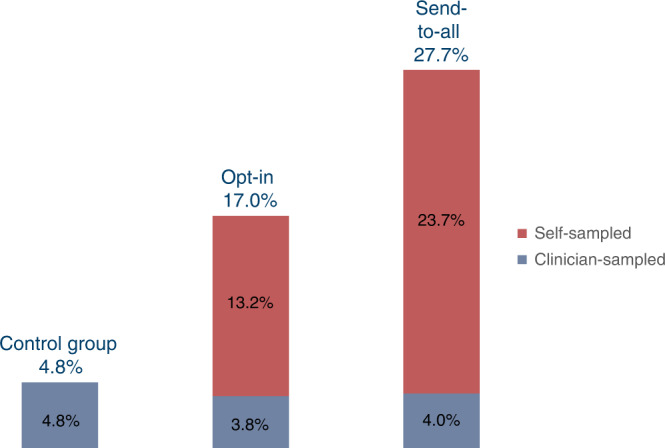
Participation rate (%) during 6 months following the invitation. Total participation was 4.8% in the control arm, 17.0% in the opt-in arm and 27.7% in the send-to-all arm.

Total participation (i.e., self-sampled and clinician-sampled tests for the self-sampling arms) was 4.8% in the control arm, 17.0% in opt-in arm and 27.7% in send-to-all arm (*P* < 0.0001, Fig. 2 and Table 2a). Thus, absolute participation differences and relative participation differences in total participation was: 12.3% (95% CI 10.3–14.2) and 3.6 (95% CI 2.9–4.5), respectively for opt-in vs. controls; 22.9% (95% CI 20.7–25.2) and 5.8 (95% CI 4.7–7.2), respectively, for send-to-all vs. controls; and 10.7% (95% CI 8.0–13.3) and 1.6 (95% CI 1.4–1.8), respectively, for send-to-all vs. opt-in (Table 2b). Differences in participation between intervention arms were largely due to self-sampling use, since there was similar attendance to clinician-collected screening: 4.8% for the control arm, 3.8% for opt-in, and 4.0% for send-to-all (*P* = 0.33, Table 2a). Per protocol analyses of participation differences are shown in Supplementary Table 1.

**Table 2:** (a) Screening participation among long-term non-attenders by intervention arm. (b) Total screening participation differences between intervention arms (intention-to-treat analyses).

(a)		Control		Opt-in				Send-to-all			
		*N*^a^	Clin. sample, *n* (%)^b^	*N*^a^	Self- sample *n* (%)^b^	Clin. sample, *n* (%)^b^	Total *n* (%)^b^	*N*^a^	Self- sample, *n* (%)^b^	Clin. sample, *n* (%)^b^	Total *n* (%)^b^
Total		1892	90 (4.8)	1897	250 (13.2)	73 (3.8)	323 (17.0)	1878	445 (23.7)	75 (4.0)	520 (27.7)
Age group											
36–45		435	35 (8.0)	433	63 (14.5)	17 (3.9)	80 (18.5)	450	121 (26.9)	27 (6.0)	148 (32.9)
46–55		497	23 (4.6)	519	66 (12.7)	23 (4.4)	89 (17.1)	504	122 (24.2)	21 (4.2)	143 (28.4)
56–65		662	22 (3.3)	653	91 (13.9)	24 (3.7)	115 (17.6)	646	141 (21.8)	20 (3.1)	161 (24.9)
66–69		298	10 (3.4)	292	30 (10.3)	9 (3.1)	39 (13.4)	278	61 (21.9)	7 (2.5)	68 (24.5)
Time since last screening test											
10–15 years		598	52 (8.7)	589	101 (17.1)	34 (5.8)	135 (22.9)	599	165 (27.5)	29 (4.8)	194 (32.4)
16–28 years		650	26 (4.0)	658	92 (14.0)	22 (3.3)	114 (17.3)	642	152 (23.7)	15 (2.3)	167 (26.0)
Never		644	12 (1.9)	650	57 (8.8)	17 (2.6)	74 (11.4)	637	128 (20.1)	31 (4.9)	159 (25.0)

(b)	Opt-in vs. control		Send-to-all vs. control		Send-to-all vs. Opt-in	
	APD^c^ (95% CI)	RPD^d^ (95% CI)	APD^c^ (95% CI)	RPD^d^ (95% CI)	APD^c^ (95% CI)	RPD^d^ (95% CI)
Total	12.3 (10.3, 14.2)	3.6 (2.9, 4.5)	22.9 (20.7, 25.2)	5.8 (4.7, 7.2)	10.7 (8.0, 13.3)	1.6 (1.4, 1.8)
Age group						
36–45	10.4 (6.0, 14.9)	2.3 (1.6, 3.3)	24.8 (19.8, 29.9)	4.1 (2.9, 5.8)	14.4 (8.7, 20.1)	1.8 (1.4, 2.3)
46–55	12.5 (8.8, 16.3)	3.7 (2.4, 5.8)	23.7 (19.4, 28.1)	6.1 (4.0, 9.4)	11.2 (6.1, 16.3)	1.7 (1.3, 2.1)
56–65	14.3 (11.1, 17.5)	5.3 (3.4, 8.3)	21.6 (18.0, 25.2)	7.5 (4.9, 11.6)	7.3 (2.9, 11.8)	1.4 (1.1, 1.8)
66–69	10.0 (5.6, 14.4)	4.0 (2.0, 7.8)	21.1 (15.7, 26.6)	7.3 (3.8, 13.9)	11.1 (4.7, 17.5)	1.8 (1.3, 2.6)
Time since last screening test						
10–15 years	14.2 (10.1, 18.3)	2.6 (2.0, 3.6)	23.7 (19.3, 28.1)	3.7 (2.8, 4.9)	9.5 (4.4, 14.5)	1.4 (1.2, 1.7)
16–28 years	13.3 (10.1, 16.6)	4.3 (2.9, 6.5)	22.0 (18.3, 25.7)	6.5 (4.4, 9,7)	8.7 (4.2, 13.1)	1.5 (1.2, 1.9)
Never	9.5 (6.9, 12.2)	6.1 (3.4, 11.1)	23.1 (19.6, 26.6)	13.4 (7.5, 23.8)	13.6 (9.4, 17.7)	2.2 (1.7, 2.8)

^a^Number of women invited.

^b^Number (%) of women screened, among women invited.

^c^Absolute participation difference, i.e. percentage point participation difference in screening by any mode (intention-to-treat).

^d^Relative participation difference, i.e. relative risk of participation in screening by any mode (intention-to- treat).

In the opt-in arm, 403 out of 1897 (21.2%) women ordered a self-sample, of which 144 (35.7%) ordered after receiving a reminder, and 250 (13.2%) returned the self-sample for analysis. Opt-in orders were made by ordinary mail (51.6%), web (33.0%), e-mail (14.2%) or telephone to the study centre (1.2%; not presented as an option in the invitation letter). Among 445 women in the send-to-all arm who submitted a self-sample, 254 (57.1%) did so after receiving a reminder.

Self-sampling increased total participation in all age groups and screening history categories. Total participation was consistently highest in the send-to-all arm, intermediate in the opt-in arm and lowest in the control arm (Table 2a). In each arm, total participation was highest in the youngest age group (age 36–45 years), at 8.0%, 18.5% and 32.9% among controls, opt-in and send-to-all, respectively, and tended to decrease with age (P-trend in total participation by age group: 0.001, 0.15 and 0.002 for controls, opt-in and send-to-all, respectively). Participation generally decreased with increasing time since last screening test, and this pattern was evident for both clinician sampling and self-sampling, and in each intervention arm (P-trend in total participation by time since last screening test (10–15 years, 16–28 years or never screened): <0.0001, <0.0001 and 0.004 for controls, opt-in and send-to-all, respectively). Among women who never had been screened previously, total participation was 1.9% for controls, 11.4% for opt-in and 25.0% for send-to-all (*P* < 0.0001). Also, the RPD was highest among never-screeners, at 6.1 (95% CI 3.4–11.1), 13.4 (95% CI 7.5–23.8) and 2.2 (95% CI 1.7–2.8) for opt-in vs. controls, send-to-all vs. controls and send-to-all vs. opt-in, respectively (Table 2b).

Among all women who were tested for HPV, 11.0% were positive for any hrHPV. The hrHPV positivity rate was slightly higher for self-sampled tests than for clinician-collected tests, at 11.5% and 9.2%, respectively (*P* = 0.40). Among controls, 6.0% were positive for any hrHPV, which was non-significantly lower than observed among women in the opt-in (10.8%) and send-to-all (11.8%) intervention arms (*P* = 0.28) (Table 3).

**Table 3:** Proportion of hrHPV at screening^a^ among long-term non-attenders in total and by intervention arm.

		Control				Opt-in				Send-to-all			
Type of HPV screening test	*N*	*N*	Any HR positive^b^, *n* (%)	16/18 positive^c^, *n* (%)	Other HR positive^d^, *n* (%)	*N*	Any HR positive^b^, *n* (%)	16/18 positive^c^, *n* (%)	Other HR positive^d^, *n* (%)	*N*	Any HR positive^b^, *n* (%)	16/18 positive^c^, *n* (%)	Other HR positive^d^, *n* (%)
Total	913	84	5 (6.0)	0 (0.0)	4 (4.8)	314	34 (10.8)	11 (3.5)	23 (7.3)	515	61 (11.8)	27 (5.3)	33 (6.4)
Clin-sample	218	84	5 (6.0)	0 (0.0)	4 (4.8)	64	7 (10.9)	2 (3.1)	5 (7.8)	70	8 (11.4)	3 (4.3)	4 (5.7)
Self-sample	695	–	–	–	–	250	27 (10.8)	9 (3.6)	18 (7.2)	445	53 (11.9)	24 (5.4)	29 (6.5)

^a^Women who attended clinician screening with a primary cytology test (*n* = 20) are not included. Six of these were in the control group, nine in the opt-in group and five in the send-to-all group.

^b^Positive for one or more of the hrHPV types 16, 18, 31, 33, 35, 39, 45, 51, 52, 56, 58, 59, 66 or 68. Two women (one control and 1 send-to-all) were registered as any hrHPV-positive, but lacked information on hrHPV-type.

^c^Positive for HPV16 and/or 18, and may be positive for other hrHPV types.

^d^Positive for one or more of the hrHPV types 31, 33, 35, 39, 45, 51, 52, 56, 58, 59, 66 or 68, but negative for 16/18.

Triage attendance was similar in the opt-in and send-to-all arms, at 77.8% and 79.3% (*P* = 1), respectively, for scheduled attendance, and 92.6% and 92.5% (*P* = 1), respectively, for any attendance within 6 months (Table 4). Attendance for women allocated to gynaecologist triage (irrespective of self-sampling intervention arm) for scheduled and any attendance was 75.6% and 90.2%, respectively, which was non-significantly lower than observed among women allocated to GP triage, who had corresponding attendance of 82.1% and 94.9% (*P* = 0.67 for scheduled attendance, *P* = 0.72 for any attendance, Table 4). The median (interquartile range (IQR)) period between a positive self-sample and the scheduled triage appointment was shorter for women allocated to GP triage than gynaecologist triage, at 19 (IQR 14, 25) and 43 (IQR 29, 54) days (*P* < 0.0001), respectively. However, among women who were biopsied and had a histology result, the median (IQR) period between the positive self-sample and the histological diagnosis was longer for women who were allocated to and attended GP triage than for women who were allocated to and attended gynaecologist triage, at 4 (IQR 3, 8) and 3 (IQR 2, 3) months, respectively (*P* = 0.01). Among women who screened clinically (control, opt-in clinician sample, send-to-all clinician sample), and who had a positive reflex triage result leading to direct referral to colposcopy with biopsy or a new HPV test after 12 months (Supplementary fig 1), 11 out of 12 (91.7%) attended follow-up as recommended by the CervicalScreen Norway (Table 4).

**Table 4:** Attendance to follow-up after a positive screening (opt-in/send-to-all self-sample) or triage test (opt-in/send-to-all clinician sample, and control).

	Opt-in self-sample, *n* (%)	Opt-in clinician sample, *n* (%)	Send-to-all self-sample, *n* (%)	Send-to-all clinician sample, *n* (%)	Control, *n* (%)
Total					
Positive tests^a^	27 (100.0)	4 (100.0)	53 (100.0)	5 (100.0)	3 (100.0)
Attended triage^b^	21 (77.8)		42 (79.3)		
Attended triage^c^	25 (92.6)		49 (92.5)		
Attended surveillance/management in CervicalScreen Norway^d^		4 (100.0)		4 (80.0)	3 (100.0)
Allocated gynaecologist^e^					
Positive tests	12 (100.0)		29 (100.0)		
Attended triage^b^	10 (83.3)		21 (72.4)		
Attended triage^c^	11 (91.7)		26 (89.7)		
Allocated GP^e^					
Positive tests	15 (100.0)		24 (100.0)		
Attended triage^b^	11 (73.3)		21 (87.5)		
Attended triage^c^	14 (93.3)		23 (95.8)		

^a^Screening-positive hrHPV self-sample, or triage positive reflex test with recommended follow-up within 12 months (control, and opt-in/send-to-all clinician sample, see supplementary Fig 1). Follow-up status for women who were recommended a new HPV test after 24 months (4 opt-in clinician sample, 3 send-to-all clinician sample, 3 control) was unavailable because it exceeded the follow-up time available by the end of the study.

^b^Attended triage within 6 months of hrHPV-positive self-sample, at allocated physician.

^c^Attended triage within 6 months of hrHPV-positive self-sample, regardless of where the exam took place.

^d^Attended follow-up within 12 months as recommended by the CervicalScreen Norway (supplementary Fig 1), i.e. repeat HPV test after 12 months, or direct referral to colposcopy with biopsy (opt-in/send-to-all clinician sample and control only).

^e^Only women with a hrHPV-positive self-sample were allocated to primary gynaecologist/GP triage.

A total of 1, 12 and 20 prevalent cases of CIN2+ were detected in the control, opt-in, and send-to-all arms, respectively (Table 5). Thus, we detected CIN2 + in 0.1%, 0.6% and 1.1% of the invited women (*P* = 0.0002), and in 1.1%, 3.7% and 3.8% of the screened women (*P* = 0.52) in the control, opt-in, and send-to-all arms, respectively. A total of 0, 4 and 7 cases of cervical cancer were detected in the control, opt-in and send-to-all arms, respectively, giving corresponding detection rates per woman screened of 0%, 1.2% and 1.3% (*P* = 0.81). In each of the self-sampling arms, the detection rate of CIN2+ and cancer per woman screened was similar among women who screened by self-sampling and women who attended screening at a clinic (Table 5).

**Table 5:** Histologically verified high-grade lesions^a^ among long-term non-attenders by intervention arm.

	Control, *n* (%)	Opt-in, *n* (%)			Send-to-all, *n* (%)		
	Clin-sample	Self-sample	Clin-sample	Total	Self-sample	Clin-sample	Total
Invited	1892			1897			1878
Screened	90 (4.8)	250 (13.2)	73 (3.8)	323 (17.0)	445 (23.7)	75 (4.0)	520 (27.7)
Had histology result^b^	3 (0.2)	15 (0.8)	5 (0.3)	20 (1.1)	32 (1.7)	6 (0.3)	38 (2.0)
Had histology result^c^	3 (3.3)	15 (6.0)	5 (6.8)	20 (6.2)	32 (7.2)	6 (8.0)	38 (7.3)
CIN2 + detected^b^	1 (0.1)	9 (0.5)	3 (0.2)	12 (0.6)	17 (0.9)	3 (0.2)	20 (1.1)
CIN2 + detected^c^	1 (1.1)	9 (3.6)	3 (4.1)	12 (3.7)	17 (3.8)	3 (4.0)	20 (3.8)
CIN3 + detected^b^	1 (0.1)	7 (0.4)	3 (0.2)	10 (0.5)	17 (0.9)	3 (0.2)	20 (1.1)
CIN3 + detected^c^	1 (1.1)	7 (2.8)	3 (4.1)	10 (3.1)	17 (3.8)	3 (4.0)	20 (3.8)
Cervical cancer detected	0 (0.0)	3 (1.2)	1 (1.4)	4 (1.2)	6 (1.3)	1 (1.3)	7 (1.3)

^a^CIN2, CIN3, ACIS or cancer.

^b^Per cent of women invited.

^c^Per cent of women screened.

A relatively high proportion of women with screening-positive tests were subsequently diagnosed with CIN2+ (Table 6). Although the percentage of histologic diagnoses was higher in the group attending gynaecologist triage, the CIN2+ yield was similar in this group when compared to women attending GP triage and women who were screened in a clinic or were triaged outside the study, at 32.3%, 34.4% and 35.3% of the screening-positive tests, respectively. A similar occurrence was observed for CIN3+ , thus few of the histologically verified cases were CIN2, an equivocal diagnosis of dysplasia [25]. Cancer was diagnosed in four women who attended gynaecologist triage, four women who attended GP triage, and three women who attended screening at a clinic or triage outside the study, which constituted 12.9%, 12.5% and 8.8% of the screening-positive tests, respectively. For all screening and triage modes, the CIN2+ yield was considerably higher among women who tested positive for HPV16/18 than among women who were positive for other hrHPV types. Among women who screened positive for HPV16/18, the percentage of CIN2+ was 64.3%, 55.6% and 69.2% for women attending gynaecologist triage, GP triage and women who attended screening at a clinic or triage outside the study, respectively (Table 6).

**Table 6:** Histologically verified high-grade lesions (*n*, % of total) among screening test positive^a^ women by mode of follow-up.

	Women screened by self-sampling and triaged by gynaecologist^b^			Women screened by self-sampling and triaged by GP^c^			Women screened by clinician (any arm) or triaged outside study^d^			
	Total positive	16/18 positive^e^	Other HR positive^f^	Total positive	16/18 positive^e^	Other HR positive^f^	Total positive^g^	16/18 positive^e^	Other HR positive^f^	Cytology positive^h^
Total	31 (100.0)	14 (100.0)	17 (100.0)	32 (100.0)	9 (100.0)	23 (100.0)	34 (100.0)	13 (100.0)	17 (100.0)	2 (100.0)
Had histology result^i^	26 (83.9)	14 (100.0)	12 (70.6)	13 (40.6)	5 (55.6)	8 (34.8)	21 (61.8)	10 (76.9)	7 (41.2)	2 (100.0)
CIN2 + detected^j^	10 (32.3)	9 (64.3)	1 (5.9)	11 (34.4)	5 (55.6)	6 (26.1)	12 (35.3)	9 (69.2)	1 (5.9)	2 (100.0)
CIN3 + detected^k^	9 (29.0)	8 (57.1)	1 (5.9)	10 (31.3)	5 (55.6)	5 (21.7)	12 (35.3)	9 (69.2)	1 (5.9)	2 (100.0)
Cervical cancer detected	4 (12.9)	4 (28.6)	0 (0.0)	4 (12.5)	3 (33.3)	1 (4.3)	3 (8.8)	3 (23.1)	0 (0.0)	0 (0.0)

^a^Positive HPV test, or ASCUS + for cytology.

^b^Women from any self-sampling arm who were allocated to and attended gynaecologist triage.

^c^Women from any self-sampling arm who were allocated to and attended GP triage.

^d^Women from any intervention arm who were screened by a clinician, and women from any self-sampling arm who self-sampled but attended triage outside the study.

^e^Positive for HPV16 and/or 18, and may be positive for other high-risk HPV types.

^f^Positive for any of the high-risk HPV types 31, 33, 35, 39, 45, 51, 52, 56, 58, 59, 66 or 68, but not positive for 16/18.

^g^Two women were registered as hrHPV-positive, but lacked information on hrHPV-type.

^h^Did not have HPV screening test.

^i^Registered with a histology diagnosis in the CervicalScreen Norway/CRN databases.

^j^CIN2, CIN3, ACIS or cancer.

^k^CIN3, ACIS or cancer.

## Discussion

We show that offering HPV self-sampling to long-term non-attending women as opt-in or as send-to-all significantly increases cervical screening participation relative to the standard reminder letter, and that send-to-all gives the largest increase in participation.

Although the effect of self-sampling interventions may differ between studies due to differences in study design and screening setting, increased participation by the send-to-all self-sampling strategy is well established compared to clinician-collected screening [15, 26]. In the present study, we observed larger absolute and relative differences in participation between the send-to-all self-sampling arm and the controls than was reported in a recent meta-analysis of self-sampling [15]. Moreover, we observed significantly increased participation for the opt-in strategy, which was not found in the meta-analysis [15]. Our results regarding self-sampling participation generally coincide with randomised controlled trials that have targeted long-term non-attenders [6, 19–21] or show stratified analyses for this part of the screening population [22, 27].

Characteristics of the targeted screening population are important for the way in which self-sampling interventions may affect participation. When self-sampling is offered more widely to women who are overdue for screening, it tends to replace screening at a clinic in addition to increase overall participation [18, 22]. We observed that the clinician-collected screening uptake was only marginally lower in the self-sampling arms than in the control arm, indicating that self-sampling hardly replaces clinician-collected screening when offered exclusively to long-term non-attenders. Our observation that some long-term non-attending women offered self-sampling still preferred to be screened by a clinician, demonstrates the importance of maintaining this option even if self-sampling is implemented in the Norwegian screening programme. Overall, we report similar effects on participation as a Swedish study targeting long-term non-attenders [6]. However, the absolute increases in participation for the self-sampling interventions versus controls were considerably higher in our study, but the relative participation difference was lower, which may relate to the slightly different screening contexts experienced by long-term non-attending women in these countries. For instance, the Swedish screening programme issues far more screening reminders than the Norwegian programme and offers prescheduled screening appointments rather than an encouragement to order an appointment for screening, which in part may explain the higher coverage observed for ordinary screening in Sweden [4] and the very low participation to clinician-collected screening among long-term non-attenders [6]. On a general note, groups that respond poorly to the control intervention, i.e. the standard invitation to attend screening in a clinic, have a high potential to benefit from self-sampling in relative terms, as we can observe in the relative participation difference estimates of the never-screeners and the women in the oldest age brackets in our study population.

Although inferior to send-to-all, we found that opt-in also increased screening participation when compared to controls who received the routine reminder letter. Thus, this type of intervention should not be dismissed, although opt-in in other settings often did not improve participation beyond a routine reminder letter to screen [15]. The way in which this intervention is offered may be of particular importance for achieving increased participation. The present study results suggest that alternative ordering options that include ordinary mail and a web-solution may be considered for opt-in among long-term non-attenders, and that a reminder to order the self-sampling device may be effective. Some effect of opt-in has also been reported in intention-to-treat analyses of other randomised controlled trials [6, 22] and observational studies [28, 29] in Scandinavia.

Long-term non-attending women may be hesitant to undergo a gynaecological examination for screening purposes [30], but our study showed that the vast majority attended to examination by a physician within 6 months if they submitted a positive self-sample. High attendance to follow-up (new HPV test after 12 months or immediate colposcopy with biopsy) after a positive reflex triage test was also observed among women in the control arm and women in the self-sampling arms who screened clinically. A high attendance to triage after a positive self-sample has also been found in other settings [15]. We found that triage attendance was similar for women who were scheduled for GP and gynaecologist triage. However, some women chose to be triaged by another physician than they were allocated to in the study, and this happened slightly more often among women allocated to a gynaecologist than among women allocated to their GP. One reason for this difference could be that the gynaecologists on average were located further away from the women than their GP. Moreover, we observed a longer time lag between notification and the scheduled appointment for women allocated to a gynaecologist, which could have compelled more of these women to reschedule to a physician who could offer an earlier appointment.

The hrHPV positivity rate of the total study population of long-term non-attenders was nearly twice as high as the corresponding rate among women of the same age in the ordinary screening population [3]. In both self-sampling arms, women who chose to attend screening at a clinic and women who chose to use self-sampling had similar hrHPV positivity rates, indicating that the mode of screening chosen by long-term non-attending women is not associated with risk of infection.

Due to low numbers, the CIN2+ occurrence observed here should be interpreted with caution, especially in terms of comparisons between intervention arms or other subgroups. Among all the long-term non-attending women who were screened during the study, 3.5% and 1.2% were diagnosed with CIN2+ and cervical cancer, respectively, while the corresponding rate among women aged 34–69 years in the CervicalScreen Norway is 1.2% and 0.1% [3]. Thus, CIN2+ and cervical cancer yield among long-term non-attenders observed in this study was very high compared to the ordinary screening population, which probably reflects a real difference in risk. However, surveillance bias could also have contributed since most physicians who triaged women who had submitted a hrHPV-positive self-sample knew that they were long-term non-attenders and part of a study, and women who were triaged by a study gynaecologist underwent colposcopy at the triage visit. However, the occurrence of CIN2+ among women with a positive screening test (hrHPV or cytology) was very similar regardless of differences in diagnostic follow-up.

GP and gynaecologist triage of long-term non-attending women who had a hrHPV-positive self-sample gave similar attendance and diagnostic yield. However, for long-term non-attending women who have CIN2+ , it is important to be treated quickly, and our results clearly showed that the final diagnostics appeared earlier if women were referred directly to a gynaecologist who could perform a colposcopic biopsy at the triage visit than if they were referred to their GP for cytology triage. Furthermore, the CIN2+ and CIN3+ detection rates among long-term non-attenders with a positive screening test exceeded the risk thresholds for which colposcopy usually is considered good practice [31, 32]. Similar to findings from the general screening population [33], we found that the risk of CIN2+ and CIN3+ was higher among long-term non-attending women who tested positive for HPV16/18 than among women who tested positive for other hrHPV types. Information form partial genotyping could be used to differentiate between referral to colposcopy and cytology triage among long-term non-attending women who submit a positive self-sample. This principle is already applied for triage positive women in the CervicalScreen Norway [33, 34].

### Strengths and limitations

A strength of this study is the use of national registry data that ensures a precise definition of the study cohort and complete information on participation and diagnostics. Targeted sampling of long-term non-attenders also ensures a sufficient sample size for robust inference regarding comparisons of participation by the study arm in this part of the screening population. Another strength is the randomised design that enhances the representativeness of the study sample and the similarity of the intervention arms. The intervention arms should thus be highly comparable, and the results of the present study should have high generalisability to the population of long-term non-attenders in Norway. Furthermore, we used a self-sampling device and a PCR-based HPV DNA test that were clinically validated and gave no invalid self-samples in this study. Finally, the trial was embedded in the CervicalScreen Norway, which should make the study results relevant to a real-world scenario where self-sampling is offered as part of an organised programme. However, the invited women were informed that this was a study, which could have influenced their motivation to participate. A further limitation is the relatively low subgroup numbers regarding hrHPV positivity, attendance to triage and CIN2+ occurrence, which limits the precision of the inference that can be made regarding these secondary outcomes of the study.

## Conclusion

We conclude that opt-in and send-to-all self-sampling strategies increase screening participation among long-term non-attenders to cervical screening in Norway. If implemented as part of the screening programme, both strategies would probably improve secondary prevention of cervical cancer and thus benefit women’s health. Long-term non-attenders also have an elevated risk for hrHPV infection and high-grade cervical lesions, which highlights the large potential to improve cervical cancer prevention by increasing the screening participation of this population. The send-to-all strategy would maximise the preventive effect because it clearly increased participation the most. Maintaining an offer for clinician-collected screening will still be important, since some long-term non-attenders offered self-sampling preferred this option. We also show that management of hrHPV-positive self-samples by GP cytology triage or direct referral to colposcopy by gynaecology specialists give similar results in terms of triage attendance and diagnostic yield. Our study supports findings that direct referral to colposcopy might be the best option for hrHPV-positive women in this population, especially if they are positive for HPV16/18, because they are at a relatively high risk for sequelae and colposcopy referral gives the shortest lag to a histologically confirmed diagnosis and treatment. Colposcopy is generally a safe procedure, thus over-referral will not be a burden for the patient.

## Disclaimer

The opinions expressed by the authors are their own, and this material should not be interpreted as representing the official viewpoint of the U.S. Department of Health and Human Services, the National Institutes of Health, or the National Cancer Institute.

## Supplementary information

Supplementary figure legends

Supplementary figure

Per protocol analysis

## Data Availability

The data contains personal information, and the study participants have not consented to public data sharing. Data access requires permission from relevant Norwegian authorities.

## References

[1] Lönnberg S, Hansen BT, Haldorsen T, Campbell S, Schee K, Nygård M (2015). Cervical cancer prevented by screening: Long-term incidence trends by morphology in Norway. Int J Cancer.

[2] Haldorsen T, Skare GB, Steen R, Thoresen SO (2008). Livmorhalskreft etter ti års offentlig koordinert screening. [Cervical cancer after 10 years of nationally coordinated screening]. Tidskr NorLegefor.

[3] Cancer Registry of Norway. Annual rapport 2019, Screening Activity and Results from the National Cervical Cancer Screening Programme, [Årsrapport 2019, Screeningaktivitet og resultater fra Livmorhalsprogrammet]. 2020. https://www.kreftregisteret.no/globalassets/livmorhalsprogrammet/rapporter/arsrapport-lp/arsrapport-livmorhalsprogrammet-2019v2_sept2021.pdf [Accessed 10 Feb, 2022].

[4] Andrae B, Kemetli L, Sparen P, Silfverdal L, Strander B, Ryd W (2008). Screening-preventable cervical cancer risks: evidence from a nationwide audit in Sweden. J Natl Cancer Inst.

[5] Pedersen K, Burger EA, Campbell S, Nygård M, Aas E, Lönnberg S (2017). Advancing the evaluation of cervical cancer screening: development and application of a longitudinal adherence metric. Eur J Public Health.

[6] Elfström KM, Sundström K, Andersson S, Bzhalava Z, Thor AC, Czoul Z (2019). Increasing participation in cervical screening by targeting long-term nonattenders: randomized health services study. Int J Cancer.

[7] Cadman L, Waller J, Ashdown-Barr L, Szarewski A (2012). Barriers to cervical screening in women who have experienced sexual abuse: an exploratory study. J Fam Plan Reprod Health Care.

[8] Chorley AJ, Marlow LAV, Forster AS, Haddrell JB, Waller J (2017). Experiences of cervical screening and barriers to participation in the context of an organised programme: a systematic review and thematic synthesis. Psycho-Oncol.

[9] Leinonen MK, Campbell S, Klungsøyr O, Lönnberg S, Hansen BT, Nygård M (2017). Personal and provider level factors influence participation to cervical cancer screening: a retrospective register-based study of 1.3 million women in Norway. Prev Med.

[10] Hansen BT, Hukkelberg SS, Haldorsen T, Eriksen T, Skare GB, Nygard M (2011). Factors associated with non-attendance, opportunistic attendance and reminded attendance to cervical screening in an organized screening program: a cross-sectional study of 12,058 Norwegian women. BMC Public Health.

[11] Leinonen MK, Campbell S, Ursin G, Trope A, Nygard M (2017). Barriers to cervical cancer screening faced by immigrants: a registry-based study of 1.4 million women in Norway. Eur J Public Health.

[12] Walboomers JM, Jacobs MV, Manos MM, Bosch FX, Kummer JA, Shah KV (1999). Human papillomavirus is a necessary cause of invasive cervical cancer worldwide. J Pathol.

[13] Egawa N, Egawa K, Griffin H, Doorbar J (2015). Human papillomaviruses; epithelial tropisms, and the development of neoplasia. Viruses.

[14] Cohen PA, Jhingran A, Oaknin A, Denny L (2019). Cervical cancer. Lancet.

[15] Arbyn M, Smith SB, Temin S, Sultana F, Castle P (2018). Detecting cervical precancer and reaching underscreened women by using HPV testing on self samples: updated meta-analyses. BMJ.

[16] Arbyn M, Verdoodt F, Snijders PJF, Verhoef VMJ, Suonio E, Dillner L (2014). Accuracy of human papillomavirus testing on self-collected versus clinician-collected samples: a meta-analysis. Lancet Oncol.

[17] Nelson EJ, Maynard BR, Loux T, Fatla J, Gordon R, Arnold LD (2017). The acceptability of self-sampled screening for HPV DNA: a systematic review and meta-analysis. Sex Transm Infect.

[18] Enerly E, Bonde J, Schee K, Pedersen H, Lonnberg S, Nygard M (2016). Self-sampling for human papillomavirus testing among non-attenders increases attendance to the Norwegian cervical cancer screening programme. PLoS ONE.

[19] Broberg G, Gyrd-Hansen D, Jonasson JM, Ryd ML, Holtenman M, Milsom I (2014). Increasing participation in cervical cancer screening: offering a HPV self-test to long-term non-attendees as part of RACOMIP, a Swedish randomized controlled trial. Int J Cancer.

[20] Sultana F, English DR, Simpson JA, Drennan KT, Mullins R, Brotherton JM (2016). Home-based HPV self-sampling improves participation by never-screened and under-screened women: results from a large randomized trial (iPap) in Australia. Int J Cancer..

[21] Kellen E, Benoy I, Vanden Broeck D, Martens P, Bogers JP, Haelens A (2018). A randomized, controlled trial of two strategies of offering the home-based HPV self-sampling test to non- participants in the Flemish cervical cancer screening program. Int J Cancer.

[22] Tranberg M, Bech BH, Blaakaer J, Jensen JS, Svanholm H, Andersen B (2018). Preventing cervical cancer using HPV self-sampling: direct mailing of test-kits increases screening participation more than timely opt-in procedures - a randomized controlled trial. BMC Cancer.

[23] Norwegian Directorate of Health. National Guidelines of Gynaecological Cancer [Nasjonalt handlingsprogram med retningslinjer for gynekologisk kreft]. 3. ed. 2021. https://www.helsedirektoratet.no/retningslinjer/gynekologisk-kreft-handlingsprogram [Accessed 8 Feb, 2022].

[24] Tavassoli FA, Devilee P, editors. Pathology and genetics of tumours of the breast and female genital organs. In: WHO classification of tumours, 3rd edn. 4. Lyon: IARC Press; 2003.

[25] Tainio K, Athanasiou A, Tikkinen KAO, Aaltonen R, Hernándes JC, Glazer-Livson S (2018). Clinical course of untreated cervical intraepithelial neoplasia grade 2 under active surveillance: systematic review and meta-analysis. BMJ.

[26] Verdoodt F, Jentschke M, Hillemanns P, Racey CS, Snijders PJ, Arbyn M (2015). Reaching women who do not participate in the regular cervical cancer screening programme by offering self-sampling kits: a systematic review and meta-analysis of randomised trials. Eur J Cancer.

[27] Cadman L, Wilkes S, Mansour D, Austin J, Ashdown-Barr L, Edwards R (2015). A randomized controlled trial in non-responders from Newcastle upon Tyne invited to return a self-sample for Human Papillomavirus testing versus repeat invitation for cervical screening. J Med Screen.

[28] Lam JU, Rebolj M, Ejegod DM, Pedersen H, Rygaard C, Lynge E (2017). Human papillomavirus self-sampling for screening nonattenders: opt-in pilot implementation with electronic communication platforms. Int J Cancer.

[29] Sanner K, Wikström I, Strand A, Lindell M, Wilander E (2009). Self-sampling of the vaginal fluid at home combined with high-risk HPV testing. Br J Cancer.

[30] Aasbø G, Solbrække KN, Waller J, Tropé A, Nygård M, Hansen BT (2019). Perspectives of non-attenders for cervical cancer screening in Norway: a qualitative focus group study. BMJ Open.

[31] Castle PE, Sideri M, Jeronimo J, Solomon D, Schiffman M (2007). Risk assessment to guide the prevention of cervical cancer. Am J Obstet Gynecol.

[32] Arbyn M, Roelens J, Martin-Hirsch P, Leeson S, Wentzensen N (2011). Use of HC2 to triage women with borderline and mild dyskaryosis in the UK. Br J Cancer.

[33] Hashim D, Engesæter B, Skare GB, Castle PE, Bjørge T, Tropé A (2020). Real-world data on cervical cancer risk stratification by cytology and HPV genotype to inform the management of HPV-positive women in routine cervical screening. Br J Cancer.

[34] Arbyn M, Rezhake R, Yuill S, Canfell K (2020). Triage of HPV-positive women in Norway using cytology, HPV16/18 genotyping and HPV persistence.. Br J Cancer.

