# Supplementary material for: HPV self-sampling among long-term non-attenders to cervical cancer screening in Norway: a pragmatic randomised controlled trial
Source: Br J Cancer. 2022 Aug 23;127(10):1816–26. doi: 10.1038/s41416-022-01954-9 (PMC9643532; doi:10.1038/s41416-022-01954-9)
Supplement: Supplementary file 1 — Supplementary figure legends [file 41416_2022_1954_MOESM1_ESM.docx]

**Supplementary figure legend**

The CervicalScreen Norway screening algorithm. The flow chart includes both clinical follow up algorithm in HPV based primary screening and clinical follow up algorithm in cytology based screening.

**Supplementary table legend**

Screening participation differences between intervention arms (per protocol analyses)
