# Supplementary material for: HPV self-sampling among long-term non-attenders to cervical cancer screening in Norway: a pragmatic randomised controlled trial
Source: Br J Cancer. 2022 Aug 23;127(10):1816–26. doi: 10.1038/s41416-022-01954-9 (PMC9643532; doi:10.1038/s41416-022-01954-9)
Supplement: Supplementary file 2 — Supplementary figure [file 41416_2022_1954_MOESM2_ESM.pptx]

## Slide 1
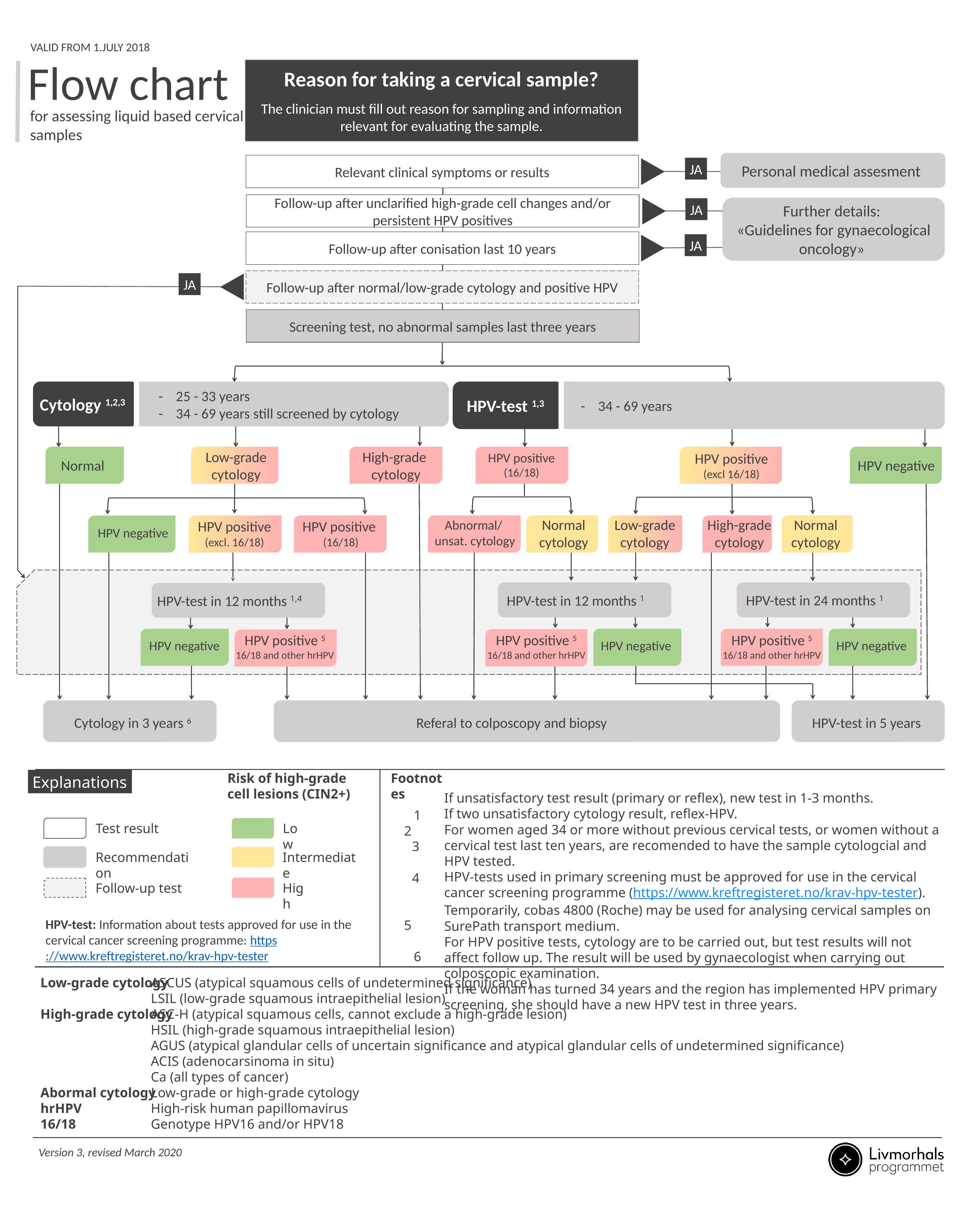

VALID FROM 1.JULY 2018
Flow chart
Reason for taking a cervical sample?
The clinician must fill out reason for sampling and information relevant for evaluating the sample.
for assessing liquid based cervical
samples
Personal medical assesment
Relevant clinical symptoms or results
JA
Follow-up after unclarified high-grade cell changes and/or persistent HPV positives
Further details:
«Guidelines for gynaecological oncology»
JA
Follow-up after conisation last 10 years
JA
Follow-up after normal/low-grade cytology and positive HPV
JA
Screening test, no abnormal samples last three years
Cytology 1,2,3
 HPV-test 1,3
 - 34 - 69 years
 - 25 - 33 years
 - 34 - 69 years still screened by cytology
Low-grade
cytology
High-grade
cytology
HPV positive
(16/18)
HPV positive
(excl 16/18)
Normal
HPV negative
Normal
cytology
Low-grade cytology
High-grade
cytology
Normal
cytology
Abnormal/
unsat. cytology
HPV positive
(excl. 16/18)
HPV positive
(16/18)
HPV negative
HPV-test in 24 months 1
HPV-test in 12 months 1
HPV-test in 12 months 1,4
HPV positive 5
16/18 and other hrHPV
HPV positive 5
16/18 and other hrHPV
HPV positive 5
16/18 and other hrHPV
HPV negative
HPV negative
HPV negative
Cytology in 3 years 6
Referal to colposcopy and biopsy
HPV-test in 5 years
Footnotes
1
 2
3
4
 5
6
Risk of high-grade cell lesions (CIN2+)
Explanations
If unsatisfactory test result (primary or reflex), new test in 1-3 months.
If two unsatisfactory cytology result, reflex-HPV.
For women aged 34 or more without previous cervical tests, or women without a cervical test last ten years, are recomended to have the sample cytologcial and HPV tested.
HPV-tests used in primary screening must be approved for use in the cervical cancer screening programme (https://www.kreftregisteret.no/krav-hpv-tester). Temporarily, cobas 4800 (Roche) may be used for analysing cervical samples on SurePath transport medium.
For HPV positive tests, cytology are to be carried out, but test results will not affect follow up. The result will be used by gynaecologist when carrying out colposcopic examination.
If the woman has turned 34 years and the region has implemented HPV primary screening, she should have a new HPV test in three years.
Test result
Low
Recommendation
Intermediate
Follow-up test
High
HPV-test: Information about tests approved for use in the cervical cancer screening programme: https://www.kreftregisteret.no/krav-hpv-tester
Low-grade cytology
High-grade cytology
Abormal cytology
hrHPV
16/18
ASCUS (atypical squamous cells of undetermined significance)
LSIL (low-grade squamous intraepithelial lesion)
ASC-H (atypical squamous cells, cannot exclude a high-grade lesion)
HSIL (high-grade squamous intraepithelial lesion)
AGUS (atypical glandular cells of uncertain significance and atypical glandular cells of undetermined significance)
ACIS (adenocarsinoma in situ)
Ca (all types of cancer)
Low-grade or high-grade cytology
High-risk human papillomavirus
Genotype HPV16 and/or HPV18
Version 3, revised March 2020
