# Supplementary material for: HPV self-sampling among long-term non-attenders to cervical cancer screening in Norway: a pragmatic randomised controlled trial
Source: Br J Cancer. 2022 Aug 23;127(10):1816–26. doi: 10.1038/s41416-022-01954-9 (PMC9643532; doi:10.1038/s41416-022-01954-9)
Supplement: Supplementary file 3 — Per protocol analysis [file 41416_2022_1954_MOESM3_ESM.docx]

**Supplementary Table: Screening participation differences between intervention arms (per protocol analyses)**

|  | Opt-in vs. Control | | Send-to-all vs. Control | | Send-to-all vs. Opt-in | |
| --- | --- | --- | --- | --- | --- | --- |
|  | **APD^1^ (95% CI)** | **RPD^2^ (95% CI)** | **APD^1^ (95% CI)** | **RPD^2^ (95% CI)** | **APD (95% CI)** | **RPD (95% CI)** |
| Total | 8.4 (6.6, 10.2) | 2.8 (2.2,3.5) | 18.9 (16.8, 21.1) | 5.0 (4.0, 6.2) | 10.5 (8.1, 13.0) | 1.8 (1.6, 2.1) |
| Age group |  |  |  |  |  |  |
| 36-45 | 6.5 (2.3, 10.7) | 1.8 (1.2, 2.7) | 18.8 (14.0, 23.7) | 3.3 (2.3, 4.8) | 12.3 (7.1, 17.6) | 1.8 (1.4, 2.4) |
| 46-55 | 8.1 (4.7, 11.5) | 2.7 (1.7, 4.3) | 19.6 (15.4, 23.7) | 5.2 (3.4, 8.0) | 11.5 (6.8, 16.2) | 1.9 (1.4, 2.5) |
| 56-65 | 10.6 (7.6, 13.6) | 4.2 (2.7, 6.6) | 18.5 (15.0, 22.0) | 6.6 (4.2, 10.2) | 7.9 (3.7, 12.0) | 1.6 (1.2, 2.0) |
| 66-69 | 6.9 (2.9, 11.0) | 3.1 (1.5, 6.1) | 18.6 (13.3, 23.9) | 6.5 (3.4, 12.5) | 11.7 (5.7, 17.7) | 2.1 (1.4, 3.2) |
| Time since last screening test | | | | | | |
| 10-15 yrs | 8.5 (4.7, 12.2) | 2.0 (1.4, 2.7) | 18.9 (14.6, 23.1) | 3.2 (2.4, 4.2) | 10.4 (5.7, 15.1) | 1.6 (1.3, 2.0) |
| Over 15 yrs | 10 (6.9, 13.0) | 3.5 (2.3, 5.3) | 19.7 (16.1, 23.3) | 5.9 (4.0, 8.8) | 9.7 (5.5, 13.9) | 1.7 (1.3, 2.1) |
| Never screened | 6.9 (4.5, 9.3) | 4.7 (2.5, 8.7) | 18.2 (14.9, 21.5) | 10.8 (6.0, 19.3) | 11.3 (7.5, 15.1) | 2.3 (1.7, 3.1) |

^1^ Absolute participation difference, i.e. percentage point participation difference in screening by clinical sample (control) and self-sample (opt-in and send-to-all) (per protocol)

^2^ Relative participation difference, i.e. relative risk of participation in screening by clinical sample (control) and self-sample (opt-in and send-to-all) (per protocol)
